# Bridging Captive and Wild Studies: Behavioral Plasticity and Social Complexity in *Theropithecus gelada*

**DOI:** 10.3390/ani11103003

**Published:** 2021-10-19

**Authors:** Elisabetta Palagi, Thore J. Bergman

**Affiliations:** 1 Unit of Ethology, Department of Biology, University of Pisa, Via Alessandro Volta 6, 56126 Pisa, Italy; 2 Natural History Museum, University of Pisa, Via Roma 79, Calci, 56011 Pisa, Italy; 3 Department of Psychology, University of Michigan, 4054 East Hall 530 Church Street, Ann Arbor, MI 48109, USA; 4 Department of Ecology and Evolutionary Biology, University of Michigan, Ann Arbor, MI 48109, USA

**Keywords:** cognitive ethology, strength/weakness of captive and wild approaches, experimental and ecological validity of the hybrid approach, reciprocal idea generators, reciprocal validation of the results

## Abstract

**Simple Summary:**

Animals are able to flexibly adjust their behavior according to both physical and social environmental changes. In this view, multiple data collections carried out in different settings are crucial to understand proximate and ultimate causes of such a behavioral plasticity. By selecting *Theropithecus gelada* as a model, we suggest that the complementary strengths and weaknesses of research in captive and wild settings makes such a hybrid approach particularly relevant. By providing information at different scales, the two settings can give a fuller picture of a behavioral trait. The apparent incongruity in behavior across captive and wild data is itself a powerful tool to explore behavioral plasticity and latent propensities. Finally, the two settings allow validating and exploring behavioral aspects that are noticed in the other settings. We really hope that our experiences and ideas can be helpful in stimulating other researchers to consider the captive-wild approach as a valid opportunity to reach a fuller picture of the behavior they are studying.

**Abstract:**

Cognitive ethology explores the ability of animals to flexibly adapt their behavior to rapid physical and social environment fluctuations. Although there is a historical dichotomy between field and captive studies, recently, a growing interest in questions that sit at the intersection of cognitive and adaptive perspectives has helped bridge this divide. By focusing on *Theropithecus gelada*, we discuss the three main reasons why this hybrid approach is extremely successful. First, captive and wild studies provide data at different social, spatial, and temporal scales that can be synthesized to give a fuller picture of the behavior. Secondly, apparently conflicting results from captive and wild settings are powerful tools to explore behavioral flexibility and latent behavioral tendencies. Third, the different settings provide ways of validating and exploring behaviors that are noticed in the other setting. Although we were able to bring together our captive and wild research to demonstrate these ideas, we could have obtained a more integrated vision on the proximate and ultimate gelada behavioral and cognitive strategies if we had considered this hybrid approach from the beginning. We hope that this manuscript stimulates scholars in designing their studies by taking into account the incredible potential of a complementary captive-wild research approach.

## 1. The Complementary Nature of Captive and Wild Studies of Cognitive Ethology

Cognition is a critical driver of how many animals respond adaptively to a changing environment. Cognitive ethology explores the ability of animals to respond flexibly and appropriately to the rapid fluctuations of the physical and social environment through the acquisition, transfer and use of information [1,2]. Although this perspective has been successful, a major difficulty in bringing an adaptive perspective to cognition (and a cognitive perspective to wild behavior) derives from different research needs. Studies of cognition benefit from experimental control while adaptive studies require ecological validity. These diverging needs created a historical dichotomy between researchers working in field and captive settings [3]. However, more recently, a growing interest in questions that sit at the intersection of cognitive and adaptive perspectives has helped bridge this divide (e.g., post-conflict behavior; social learning; animal personalities; communication) [4,5,6,7,8].

The cognitive ethology approach has created inroads from both directions. Captive work has benefited from focusing on behaviors that maintain ecological validity (e.g., the ability to recognize faces is unlikely to be present in one setting but absent in the other) while field work has benefited from incorporating greater experimental control (e.g., playback experiments). These hybrid approaches have proven enormously successful. However, we feel that there is still important information to be gained from bringing together research across both captive and field settings. The synergy between captive and wild studies appears fundamental for at least two main reasons. First, the different environmental challenges help unveil the complexity and flexibility of the behavioral cognition of animals. Essentially, the captive setting provides an (un)natural experiment to look at how animals respond to different situations. Thus, researchers can observe flexibility that can only be found by comparing across captive and natural settings. Second, and perhaps more importantly, the two settings lend themselves to different approaches that complement each other. The greater ease, control, and detail of observation in the captive setting can be used to pilot, validate, and interrogate phenomena in great detail. Conversely, the ecological validity of the wild is necessary for linking these phenomena to fitness consequences. In addition, the large scale of the wild setting (large groups, natural home ranges, intergroup interactions, habitat variation, etc.) expands the horizon of phenomena that can be observed in captivity.

One example of the complementary nature of captive and wild studies comes from the study of multimodal communication in ring-tailed lemurs (*Lemur catta*). Olfactory cues, associated with the visual ones, regulate many aspects of lemur biology and social life such as intra- and inter-sexual competition, territorial defense and dominance relationships [9,10]. Data collected on captive lemurs provided evidence about the important role of urine (highly neglected for many years) in intra-species communication [11]. In ring-tailed lemurs, urine marking is associated with a conspicuous visual cue: the tail raised up. By first making detailed observations in captivity, the authors were able to identify possibly important variations in how lemurs perform urine deposition. Then, the function of these behavioral variants was explored in the wild, showing that one form of scent marking is a multimodal signal used in inter-troop communication and territorial defense [12]. The complementary approach of captive and wild studies allowed testing both proximate (e.g., olfactory discrimination abilities in captive lemurs) and ultimate factors (e.g., territory defense in wild lemurs) at the basis of the evolution of a multi-modal signal in lemurs.

Another illustration of the complementary approach can be seen in the use of captive settings to validate methodology that is then used in wild settings. This is commonly done in the field of endocrinology. A strong form of physiological validation for novel sampling methods or assays is to administer a pharmacological manipulation that stimulates (or suppresses) a particular hormone. This is often only feasible in captivity. For example, Beehner and McCann [13] administered an ACTH challenge to captive geladas (*Theropithecus gelada*) to show that the fecal extraction method and glucocorticoid assay reliably captured a glucocorticoid spike following ACTH injection. The method was then used to study glucocorticoid variation in wild geladas in relation to altitude and seasonal variations.

Here we focus on three general themes that illustrate different complementary aspects of captive and wild studies. For each theme, we highlight our own captive and wild research in geladas as an illustration of the strength of the combined approach both retrospectively (i.e., areas where we have been able to bridge captive and wild studies) and prospectively (i.e., areas where we see potential for the combined approach to further advance our current understanding). First, captive and wild approaches naturally lend themselves to studies at different spatial, temporal, and social scales. Captive studies often enable finer scale observations and analysis that might not be possible in field conditions. Conversely, the natural settings often encompass broader scales that might not be present in captive settings. Thus, while captive settings might be better for uncovering the intricacies of particular social interactions, wild studies can look at multi-group dynamics across scales that do not exist in captivity. To illustrate how working at both scales can lead to a fuller understanding of behavior, we focus on the use of social information in geladas. The second theme concerns how wild and captive studies often reveal different behaviors. While this conflict can be confusing and difficult to interpret, it can also reveal flexible aspects of behavior, latent tendencies, and the ability to respond to external changes. Here we focus on an exploration of novel objects by geladas as a way to illustrate this phenomenon. As a third step, captive and wild settings differ in their level of experimental control. Captive settings have relatively high control and low ecological validity, while the reverse is true in the wild. Therefore, different aspects/stages of the research process are favored. Ideas can be piloted in captivity and tested in the wild, but the opposite can also be true. Using both approaches enables a more complete study program. To highlight this theme, we use studies of communication in geladas and anticipate some possible aspects of research that can be addressed by integrating future data from wild and captive settings.

## 2. The Model Species: Geladas and the Study Sites

The large number of studies carried out both on wild and captive populations of geladas (*Theropithecus gelada*) make the species a suitable model to evaluate the complementary role of these two environmental settings in the study of animal behavior. Since the publication of the first studies on geladas (*Theropithecus gelada*), the species has appeared in all its uniqueness not only for its evolutionary history [14] and anatomy [15,16,17], but also for its ecology and behavior [18,19].

Geladas differ from other closely related taxa of papionine (e.g., *Papio*, *Mandrillus*) in several anatomical and physiological features. Due to their phylogenetic position (closely related to the mangabey genus *Lophocebus*), and ecological and behavioral distinctions, there is a growing consensus in not considering geladas as a species of baboon [20]. For example, from an ecological perspective, they are the only known graminivorous primate species [21]. In their long-term project, Dunbar and Dunbar [18] and Kawai [22] provided the first description and standardized data on the behavioral ecology and social dynamics of wild geladas. Geladas live in multi-level societies with fission–fusion dynamics [23,24]. In the wild, gelada multilevel societies have core units (first level of organization) [25,26] that consist of polygynous reproductive units (hereafter, one-male units, OMUs) and all-male units (hereafter, AMUs) that are easily discernible due to their high levels of consistency in spatial and social cohesion. Such basic groups can fission and fuse with one another across years, days and even hours in a highly plastic way. In wild geladas, AMUs tend to be peripheral and inconsistent in their association patterns while OMUs are arranged in discrete levels of organization. In the intermediate level of the organization, the team makes up individuals associated in 90% of cases and in the upper level, the band includes subjects who generally spend half of their time sleeping and foraging in proximity [27]. The apex level of organization, the community, is the largest association of units that ever encounter each other which is stable over time and differs from the herd, a term indicating a temporary association of OMUs/AMUs that can fluctuate at a daily level [24].

Although inter-unit relationships in the wild are not based on social exchange and affiliation, such a complex fluid system based on units’ specific spatial aggregation underlines the extraordinary level of inter-individual tolerance shown by the species. It has been supposed that geladas’ grass diet is at the basis of such high levels of tolerance between groups; the impossibility of monopolizing such an abundant resource leads to low levels of food competition not only at the intra-, but also at the inter-unit level [23,28]. However, the specific ecological and social factors driving cohesion at different levels of association is still unknown and it will be one of the topics to address in future wild research.

The enormous span of gelada societies may seem to render them a poor candidate for captive studies where the higher-level dynamics are completely absent. However, we feel that the large contrast between captive and wild settings makes such species particularly interesting for research that spans captive and wild observations. Any differences make the dual approach more informative and the detail available in captivity more complementary [29]. Indeed, more than ten years of data on captive groups have been providing important insights on the inter-individual dynamics within the OMU. To date, 436 out of the 466 captive geladas are hosted at the European Zoological Institutions with the most numerous colony (*n* = 93) hosted at the RheineZoo (Germany), where most of the captive studies took place (source European Endangered Species Programme, 31 December 2020; https://www.eaza.net/assets/Uploads/CCC/BPG-2021/Gelada-baboon-BPG-final-edit-070521-compressed.pdf).

## 3. Theme 1—Complementary Scales of Research: Geladas’ Use of Social Information

Behavior manifests itself on a variety of scales, and spanning these scales can be facilitated by working on both captive and wild settings. For social behavior, scales can range from dyadic interactions to group movements involving hundreds of animals in some species. While group-level behavior emerges from individual decisions, it is important to understand the causes and consequences of behavior on both narrow and broad scales. This is often difficult to do in a single research setting—the observation conditions for large-scale phenomena often preclude a more fine-grained observation. Conversely, settings that promote fine-scale observation often may not exhibit the large-scale phenomena. In general, captive settings facilitate fine-scale analyses, while field settings are often necessary to explore broader phenomena. By bringing both perspectives together, it is possible to thoroughly document the phenomena across a range of scales. We illustrate this by focusing on geladas’ use of social information.

Social information is important for navigating complex social worlds. It is particularly important in tolerant, flexible societies where power is widely distributed and the dominance slope is weak [30,31]. In such societies, animals need to continuously negotiate their social interactions, which are not strictly codified by rank rules [30]. The need to remove uncertainty in social information is particularly important when we consider the complex ways in which individuals interact with multiple individuals simultaneously [32,33]. Focusing on triadic interactions allows an understanding of if/how third subjects perceive and interpret relationships between others (e.g., agonistic support, consolation) and if/how others are aware of how their own actions are perceived or not by third parties (e.g., tactical concealment). In this view, triadic awareness (*sensu* de Waal [34]) modifies the behavior of third subjects according to the social world in which they operate [35]. Triadic awareness can improve social integration because individuals, in the long term, become expert in coping with new and unexpected social situations that are extremely frequent in those societies and are characterized by a certain degree of social tolerance [36].

**Figure 1:**
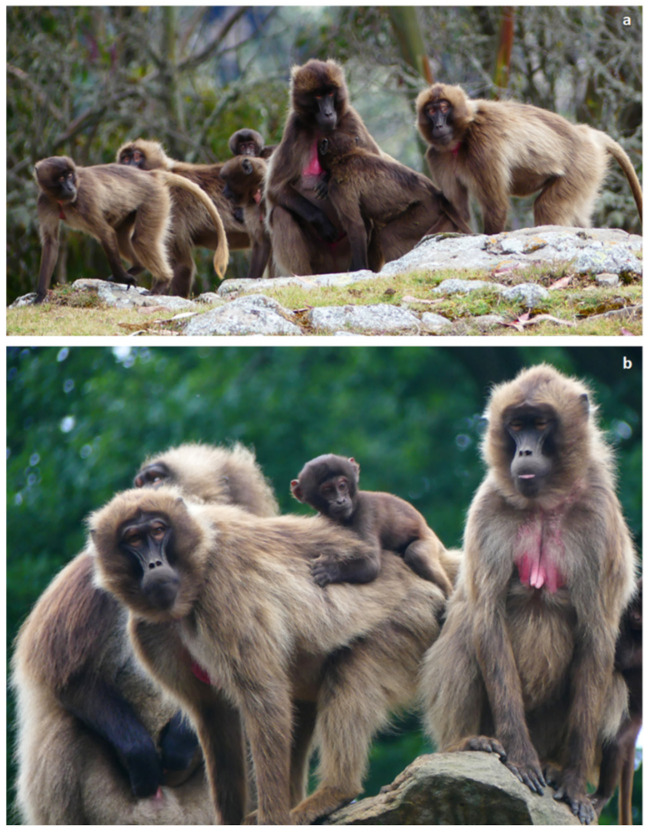
A group of adult females and immature subjects of geladas (*Theropithecus gelada*) (**a**) from the Kundi plateau in the Wof-Washa area (Amhara region, Ethiopia) and (**b**) from the NaturZoo (Rheine, Germany). Photo: Elisabetta Palagi.

Geladas offer a unique opportunity to explore the role of triadic interactions in the negotiation of relationships at different levels of their social structure. Both in the wild and in captivity, the gelada core unit (one-male unit, OMU) is a “social microcosm” where both the alpha males and females are socially integrated [28,37,38]. In contrast to hamadryas OMUs, gelada females, representing the philopatric sex [23,28], show a linear maternally inherited dominance hierarchy that does not reach the same degree of power asymmetry as that of other Old World monkeys [28,39] (Figure 1). The low dominance steepness characterizing gelada females probably has roots in the geladas’ feeding ecology. As mentioned above, the difficulty in monopolizing widely distributed food resources (i.e., grass) leads to a scramble for competition which, in turn, translates into more relaxed and tolerant relationships [40,41].

### Examples of the Role of Triadic Interactions in Geladas Come from Both Captive and Wild Populations

In an aggressive context, in addition to the two opponents, several subjects can directly interact. The possibility of precisely monitoring the subjects involved in each aggressive event can provide interesting data on the role of third parties not only after but also during an ongoing agonistic contact. Understanding whether triadic interventions are driven by contingent social situations (*hic et nunc* decision making) or are planned by individuals to respond to long-term strategies (*a priori* decision making) is required to explore the cognitive abilities at the basis of triadic awareness.

A third subject can reduce the arousal of two potential opponents well before the eruption of aggression, thus making clear that animals are able to anticipate conspecifics’ behavior. Under captive conditions, it is common to observe immature and adult geladas’ approaching, lip smacking and grooming a subject who is threatening a groupmate. Immediately after such appeasement interactions, the potential aggressor calms down and starts to exchange grooming with the “peacemaker” (Pallante and Palagi, anecdotal observation in captivity). In geladas’, calming behaviors can be offered also after the end of the conflict in the so-called unsolicited third-party post-conflict affiliation [42]. Pre- and post-conflict interventions are not randomly distributed, but individuals selectively offer support or affiliative contacts to specific subjects and under particular circumstances. Such selectivity indicates that third subjects are aware of the long-term relationship shared between the two opponents (e.g., kinship, rank distance, bonding) and of the contextual factors immediately affecting the conflictual situation (e.g., proximity of a relative, intensity of aggression).

The flexibility in the tactical interventions by third parties can open a window on the social competence and cognitive skills of subjects living in complex social groups. Immediately after an aggression, captive geladas (NaturZoo, Rheine, Germany) spontaneously offer comforting gestures to the victim especially when the conciliatory contact between the former opponents has not yet taken place [38]. The comforting gestures involve short play bouts (mainly offered by immature subjects), touching, embracing and facial expressions and vocalizations (lip-smacking/grunting and moan). The preference for such rapid behavioral patterns could be due to the level of arousal characterizing the minutes immediately following the conflict. Despite their rapidness, triadic contacts provide important direct benefits to the victim; indeed, they seem to a have a direct role in reducing anxiety (i.e., a strong decrease in self-directed behaviors) and the probability of renewed aggression. Interestingly, third parties do not generally concentrate their affiliative contacts on either kin or friends, thus suggesting that such post-conflict interventions are probably aimed at maintaining stability and cohesion at the group level. The role played by the highest-ranking members in providing post-conflict affiliation seems to corroborate the group stability hypothesis. Indeed, the affiliative gestures offered by high-ranking third parties is generally most effective in reducing the likelihood of renewed aggression towards the victims, as well as their level of anxiety [38].

The agonistic support provided by third parties to one of the two opponents during intra-OMU ongoing conflicts can be a good model to analyze which is a possible evaluation at the basis of the decision to intervene. Pallante et al. [39] found that in captive geladas the victims received more agonistic support than the aggressors. While the agonistic support directed towards the aggressors was randomly distributed, the agonistic support provided to the victim was more goal-directed. High-ranking subjects provided agonistic support mainly to the lowest-ranking victims with the immediate effect to reduce the probability of a reiteration of the attack thus suggesting that the strategy is effective in the maintenance of social homeostasis. Interestingly, both the alpha male and high-ranking females were active in supporting the victims. This result obtained from the captive population agrees with data coming from the population of the Simien National Park (Ethiopia). In his early work on the social behavior of the species, Dunbar [43,44] underlines the importance of the female alliances independently from the alpha male that intervenes only when the females involved in a conflict lack female supporters.

**Figure 2:**
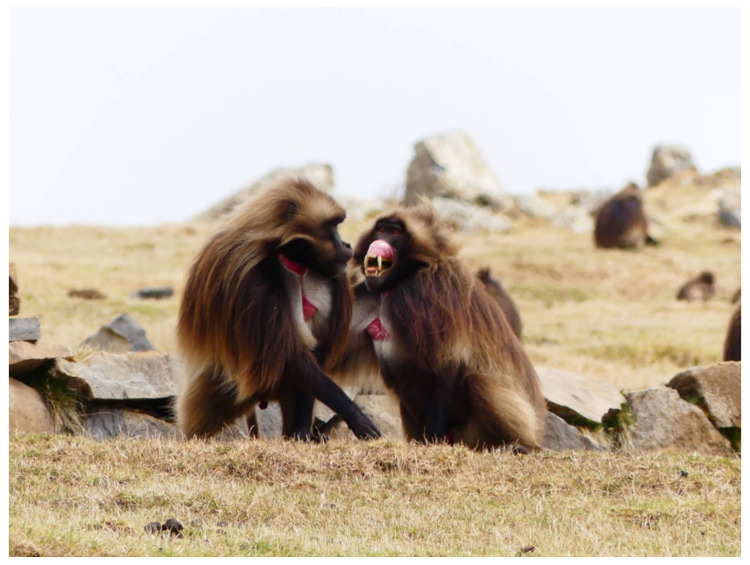
Post-conflict interaction between two adult males belonging to a bachelor group (Amhara region, Ethiopia). The subject on the right side is performing a lip-flip, a facial expression indicating benign intent. Photo: Elisabetta Palagi.

Data from captive geladas indicate that the potential supporters can evaluate whether helping a victim is convenient or not. Geladas tend to intervene more when the victim does not share either strong bonds or kin relation with the previous aggressor thus suggesting that, by monitoring the behaviors of group members, animals can get information about their relationship. Two weakly bonded or unrelated opponents are generally less likely to engage in conciliatory contacts (i.e., reconciliation, Figure 2) [37] that represent the most effective post-conflict mechanism in limiting the risk of subsequent aggression [45]. In this view, the triadic awareness, unveiled by the capacity of third subjects to modulate their behavior as a function of the relationship quality linking aggressors and victims, has direct implications for the maintenance of the cohesion within the OMU, a phenomenon also known as ‘community concern’ [46].

To date, work with wild geladas has focused on the extent of social knowledge and understanding how geladas make social decisions when navigating their enormous societies. Different from other monkeys organized in multilevel societies containing about 150 individuals (e.g., *Papio hamadryas*) [47], gelada communities may contain more than 1000 individuals [27,48]. Such large societies pose obvious challenges to the members as they are unlikely to have detailed information about each member of the community. Therefore, it can be difficult to know who might make an easy target for an attack, who is a potential mate, or who is a potential ally.

Bachelor males face challenges in assessing rivals and choosing mates because they are the ones who initiate challenges to the reproductive units [49]. How do they decide which males to attack? Given the costs of the sometimes lethal takeover fights, choosing weak rivals or willing mates can have enormous fitness benefits. One idea is that bachelors might use triadic information about the relationships between particular males and females. Males with weakly bonded females may be easier to oust from a unit as females can side with challenger males and decide the outcome of takeover fights. Therefore, monitoring the relationship status within the dozens of units (and hundreds of individuals) they may encounter in a day would be beneficial, although quite cognitively challenging. However, playback experiments found no evidence of this kind of monitoring [50]. Simulated copulations between males and females (by playing overlapping copulation calls) found no differences in response to ‘normal’ copulations (between males and females in the same unit) and surprising copulations (between males and females in different units, something that almost never occurs). This result contrasts with chacma baboons where males respond strongly to simulations that indicate disruptions of even temporary consortships between other males and females [51]. Thus, it seems that gelada bachelors do not recognize (at least vocally) which males and females belong together in the same unit, which would be necessary for monitoring their relationship quality [49].

These findings from bachelors fit with studies of the use of social information in leader males. Bergman [52] tested the limits of vocal recognition in leader males by simulating the approaches of males by playing non-threatening vocalizations (grunts) from behind a visual barrier 5–10 m away. Leader males responded to males with intermediate social overlap (members of their own band but not their unit or team) the same way they do to completely unknown males. Only males within their own unit (and possibly team--small samples sizes preclude accurate assessment here) seemed to be vocally recognized, meaning that wild geladas are in the very unusual situation for a non-human primates of having frequent contact with strangers [52].

Given that wild geladas seem to be limited in their use of social information, how do they navigate their societies? We have found evidence that they use a combination of simple cues and sexually selected signals [53]. For example, bachelor males attend to fights between other males and recent takeovers as these likely indicate opportunities for surreptitious mating [49]. Furthermore, unit males have both a visual signal (a bright red chest patch) and a vocal signal (the display call) that correlate with condition [54,55]. Therefore, bachelors can attend to these signals and assess males that they do not recognize allowing accurate assessment in the absence of social knowledge [56].

However, it is not the case that wild geladas avoid social information altogether. Zooming in on interactions within reproductive units reveals considerable evidence for sophisticated social monitoring. For example, the rare but observable copulations of subordinate follower males reveal evidence for tactical concealment [50]. From an operational point of view, tactical deception occurs when a subject uses a behavioral pattern (actions or signals) typical of its repertoire in an atypical context to misinform groupmates [57]. The opportunistic modulation of the behavior includes the falsification and/or the suppression of specific signals to preclude the possibility for some groupmates to perceive such signals and use them to their own advantage. In wild geladas, during extra-pair copulations, both males and females strategically inhibit their copulation calls, presumably to avoid being detected by the “cuckolded” male, which can aggressively punish the copulating dyad [50]. Furthermore, the animals modify their calls according to the variability in the detection risk as measured by their distance to the dominant male. Therefore, wild geladas are likely paying very close attention to the behaviors and interactions of other members of their immediate social group.

In sum, combining captive and field studies of gelada use of social information gives a fuller picture of gelada social cognition. Looking at just the field and larger scale breadth questions suggests that geladas are relatively limited in their use of social information as they fail to even recognize other members of their own band. However, captive studies show a rich and sophisticated understanding of other individuals and their relationships and dynamic interactions. Rather than being in conflict, these differences reinforce the idea that animals have different levels of detail in their social knowledge depending on the scale of interaction. Within reproductive units, geladas are clearly using sophisticated social cognition, which also coincides with the use of tactical deception in the wild. A single dimension, such as the number of individuals recognized, cannot give a full picture of how animals use social information.

For social information, field studies are particularly useful for breadth questions and captive studies are particularly useful for depth questions. Only when combining information on the breadth and depth of the use of social information do we understand its importance to the animals.

## 4. Theme 2—The Value of Variation across Field and Captive Settings: Gelada Novel Object Exploration

Often, we observe behavioral differences across captive and wild settings (e.g., variation in object manipulation and neophilia in wild and captive orangutans) [29]. Indeed, it is precisely these differences that can make captive settings unpredictable for studying questions related to adaptive behavior. It is difficult to even know if what is seen in captivity would also exist in natural conditions. However, this variation also has at least two distinct advantages. First, the variation is, in itself, an indicator of the flexibility of animals. Different captive settings amount to experiments that can be used to test the influence of various physical and social factors on behavior. Second, captivity can reveal latent tendencies (e.g., because of relaxed time pressure) that are not apparent in the wild. The mere existence of the behavior can reveal potential plasticity and flexibility that might help us understand past and future behavior.

Captivity can be particularly important for revealing behavioral innovations. For example, tool use in primates tends to be more common and more elaborate in captivity [58]. Not only do observation conditions in captivity increase the chances of detecting novel behaviors, but the captive environment might also be conducive to generating such behaviors. For example, direct comparisons show that captive orangutans are clearly more neophilic than wild orangutans, which could facilitate innovation [29]. Indeed, captivity has revealed a latent tendency for vocal imitation in orangutans that goes beyond anything seen in the wild [59]. For an innovative behavior to emerge and develop in the repertoire of a group of animals, the costs associated with the enactment of the new behavioral pattern should be lower than the incurred advantages in terms of fitness. Under different environmental conditions (e.g., captive and wild settings) this pay off can vary with captivity generally being associated with lower costs of exploration because of relaxed time and energetic constraints due to provisioning. In such cases, animals are able to flexibly generate new behaviors by co-opting and re-arranging other patterns when they need to resiliently react to environmental changes [60].

As an example, stone handling is a behavioral innovation that appears to only emerge under relaxed feeding pressure in captive or provisioned primates. Stone handling is a form of solitary object play that ranges from simple to more complex manipulative actions that appear to be self-rewarding for the actor. The behavior has been extensively studied and well-documented in both captive and provisioned free-ranging troops of Japanese macaques (*Macaca fuscata*) where it seems to be culturally transmitted across generations [61,62,63,64]. Since stone handling has never been reported in non-provisioned wild groups of macaques [65], it seems that, at least in this *taxon*, large food availability is one of the propulsive engines for the behavior to emerge.

We have found the same pattern in geladas. Captive geladas spend a considerable amount of time in manipulating stones [61]. In the colony of geladas housed at the NaturZoo (Rheine, Germany), stone handling was detected for the first time in 2007 when only a few subjects (some juveniles and one adult male) engaged in the behavior. In the following years, almost 50% of subjects of all age-sex-classes handled the stones in a variable way, thus suggesting that, as it occurs in macaques, the behavior can be culturally transmitted also in geladas. To verify this hypothesis, we would need to collect data on different groups of captive geladas whose subjects never entered into contact with each other to check for the potential presence of the phenomenon. Moreover, if the behavioral trait is culturally transmitted, we should find different patterns forming the stone handling repertoire to be the result of group idiosyncrasies.

During a period of standardized data collection on captive geladas, Cangiano and Palagi [61] found that not all the stone handlers performed complex sequences of actions. Immature subjects manipulated the stones more frequently than adults, although adult sessions were much longer and richer in complexity than those of infants and juveniles. Creating unpredictable and rewarding situations with unfamiliar objects probably fosters the emergence and development of novel manipulative patterns that tend to acquire functional roles over time [66]. Indeed, object play is an activity which is more frequent in species whose diet relies on complex hand movements to extract nutrients from different kinds of food [60].

Stone handling behavior has not been observed in wild geladas. Indeed, wild geladas have a notable lack of interest in novel objects, scoring much lower than baboons on measures of interest in objects that they encounter [67]. While baboons often approach and handle objects like a tennis ball, geladas typically only glance at the object and continue foraging. These striking differences presumably relate to geladas relatively homogenous diet and lack of a benefit to exploring new objects at least in the wild.

The captive–wild comparison suggests that if gelada’s ecology changed to make exploration more beneficial, wild animals would be able quickly to adjust their level of interest in novel objects. Captive data also coincide with the finding that in the wild juvenile geladas show more interest in novel objects than adults [67]. The relatively low levels of curiosity and play with novel objects in wild geladas could be linked to the fact that, to satisfy their daily subsistence necessities, they need to spend a significant amount of time foraging. This obviously leaves little time for some forms of play to emerge. Hence, despite appearing relatively incurious in the wild, geladas maintain developmental and evolutionary flexibility in their propensity to explore.

## 5. Theme 3—Field and Captivity as Reciprocal Providing Grounds: Communication and Coordination in Geladas

One final way that we see captive and wild studies working together is their ability to generate in one setting and to validate in the other. Differences in observability, ecological validity, and experimental control mean that different aspects of the research process are more feasible in one setting than another. A combined approach harnesses the strength of both settings. For example, we might think of a phenomena or behavior being discovered in captivity (where closer observations might detect subtle or rare events) and then ‘validated’ in the wild. Here, validating can mean anything from documenting its existence to detailed studies of the fitness consequence of the behavior. However, the process also works in the other direction. In particular, phenomena that are observed or studied in the wild can be brought into the laboratory for more robust experimental manipulation. This direction of validation can be seen in the use of captive settings to validate endocrine methodology, as described in the introduction.

Validating and manipulating in captivity is particularly relevant in cognitive studies because similar behaviors can be achieved through very different cognitive means. For example, animals might respond to the alarm calls of others because they understand that the alarm call indicates knowledge on the part of the caller about an unseen predator (a cognitively rich explanation) or they might have a simple association between the sound and the response (a cognitively simpler explanation). Therefore, the ability to experimentally manipulate the situation is particularly important for understanding wild behaviors where the underlying cognition or motivation is of interest. To illustrate this bi-directional relationship, we focus on the communication and cognition of geladas. Note that in this case, some of the validation is prospective and has not been performed; we include it to encourage people to think about linking captive and wild studies early in their research design. Had we done this ourselves, our own research would have progressed more efficiently.

The sensory world as it is experienced by an organism (*UMWELT, sensu* von Uexküll et al. 1899) [68] is at the basis of the huge variability in signal transmission. The cue components recruited in a signal can rely on different sensory modalities (visual, olfactory, acoustic). In this multi-sensorial world, scents, body postures, facial expressions or vocalizations can be produced to transmit different messages under different situations [69]. In this section, we will describe the variability of communication in geladas and explore how their ability in transmitting and decoding different types of signals increases behavioral coordination at different scales.

The audience of a signal can be represented by the subject directly interacting with the sender (dyadic scale) and by third parties attending the interaction (triadic scale). In captivity, the possibility to closely look at the emission of the signal and the exact receiver responses can help generate ideas and hypotheses that can be later tested in the wild. The wild studies then allow to look for the presence and modality of certain communication patterns and their fitness consequences in natural settings (proximate and ultimate factors, *sensu* Tinbergen, 1963 revisited in Bateson and Laland, 2013) [70].

One of the relatively well-studied communicative domains under captive condition is physical–locomotor play, also known as play fighting [71,72]. In captive geladas, play can involve not only youngsters but also adults, especially the females that are motivated to play not only with immature subjects but also with other adult females [73]. Although adult social play is favored and amplified by captive conditions (e.g., artificial feeding, low stress level) [74], the adults of some species showing a similar social organization to geladas do not show this behavior either under captive conditions (i.e., hamadryas [75]). More recent studies have unveiled that, in many primate and non-primate species, the presence of adult play is indicative of tolerant and relaxed inter-individual relationships [76]. Interestingly, in captive geladas, adult and immature play fighting correlated with the quality of the relationship shared by the subjects and not with their level of aggressive contacts, thus suggesting that geladas engage in social play to improve their social affiliation more than to gain competitive advantages [73].

**Figure 3:**
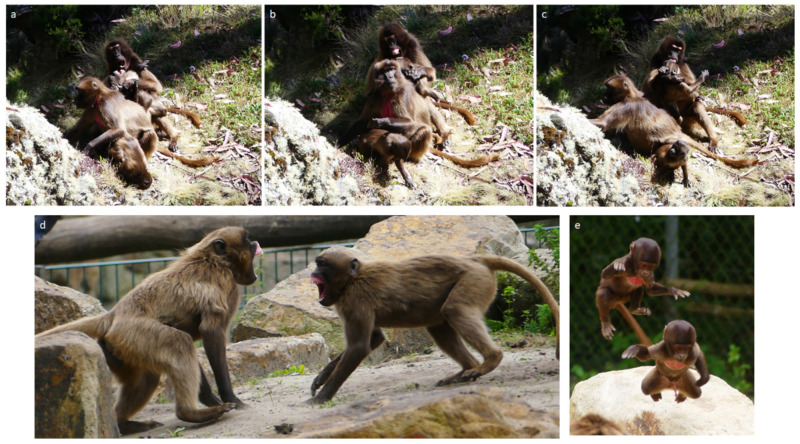
Social play in geladas. (**a**–**c**) sequences of an alloparental play fighting session involving an adult female and an immature subject (Amhara region, Ethiopia); (**d**) rapid facial mimicry of full play face between two subadult males at the NaturZoo (Rheine, Germany); (**e**) play running between two black infants at the NaturZoo (Rheine, Germany). Photo: Elisabetta Palagi.

The findings on the possible roles of play fighting in geladas have been recently validated in the wild, although the study on natural populations provided a less clear-cut picture of the phenomenon [77]. The authors found that the competitive nature characterizing play fighting in this species can change according to the group membership of the players involved. Thanks to the video-analysis of 527 play fighting sessions, the authors were able to finely evaluate the asymmetry of the play patterns (Figure 3). Although play in wild geladas does not seem to suffer social canalization being equally distributed across age, sex and group membership, play sessions between subjects belonging to different OMUs are shorter, more unbalanced and less coordinated (less role-reversal and self-restraint, more competitive play) compared to those involving same-OMU players (more role-reversal, self-restraint and cooperation, more cooperative play). Hence, in geladas, play can be a tool for both reinforcing social bonds (intra-OMU play, captive and wild studies) and improving the physical skills necessary to cope with either future mates or competitors (inter-OMU play, wild studies). Thanks to the captive-wild synergic approach, it has been possible to unveil that the different behavioral coordination and cooperation of the players can be at the basis of the multifunction role of playful activity in this species.

Gelada facial expressions are characterized by a high degree of structural blending (captive data, full play face; wild data, lip-flip, Figure 2) [78,79] and a high plasticity in the use of the same facial display across different contexts [80,81] that increase the visual communicative complexity of the species. During their play fighting, animals can recruit facial expressions to communicate their benign intent and to anticipate some offensive patterns that, thanks to the preceding facial displays, can acquire different meanings (metacommunication) [82,83]. Such metacommunicative signals have a role in managing the session and avoiding misunderstanding during the roughest sessions [84].

While playing, geladas show a variety of facial expressions; some of them are highly context-specific (play face and full play face, 78) while others can also be performed in positive contexts different from play (e.g., lip-smacking). Both wild and captive data suggest that geladas are highly attentive to each other’s faces and can frequently engage in face-to-face interactions to regulate their social contacts (e.g., play, mating [80,85]). Geladas are also able to mirror in a very fast way (<1 sec latency) others’ facial expressions, especially during their play fighting contacts [80]. The rapid facial mimicry phenomenon has a significant effect in prolonging the playful sessions and seems to be independent from the number of play faces performed during play [81]. One of the factors that appears to affect the rapidity and the rate of the mimicry response is the familiarity of the two interacting subjects [80]. Data collected in the wild should allow an understanding if the use of play faces and the rapid mimicry phenomenon can be modulated at a higher scale level according to the group membership of the playing subjects. Since play modality differs between intra- and inter-OMU players [77], we would expect that play signals can be adjusted as a function of the more cooperative or competitive nature of the interaction.

**Figure 4:**
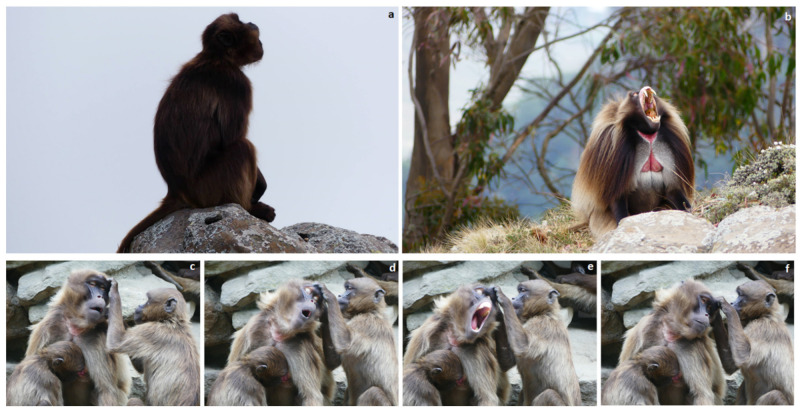
The same yawn variability is present both in captive and wild conditions. Two examples of yawning in geladas (a-b: Amhara region, Ethiopia; c-f: NaturZoo, Rheine). (**a**) yawn with covered teeth from a subadult male and (**b**) yawn with uncovered gums from an adult male. (**c**–**f**) Sequence of a yawning event performed by an adult female while receiving grooming from a juvenile female. Yawns with uncovered gums can be performed both by males and females. Photo: Elisabetta Palagi.

Moving outside the playful context, another interesting phenomenon is yawn contagion, a widespread and well-known facial mimicry phenomenon in vertebrates [86]. Notwithstanding the strong debate about the proximate factors underlining the behavior [87], there is a consensus that yawn contagion is a behavioral tool to help synchronization between subjects [88,89] thus probably leading, in the long term, to an increase in familiarity between subjects [90]. In the Rheine colony, Palagi and co-workers [90] found that geladas were infected by others’ yawns. In the long-term project, the authors were able to recognize every single subject forming each of the one-male units and to count in a highly standardized way the exact number of grooming session exchanged between subjects. The authors were able to evaluate whether social bonding significantly affected the rate of yawn contagion and they found that contagious yawning positively correlated with the frequency of the grooming exchanged between subjects, but not with their level of spatial proximity (Figure 4), thus making clear that it is not enough to stay close in space to enhance the level of yawn contagion.

More recently, thanks to the use of video-cameras, Gallo et al. [91] were able to collect standardized data on yawn contagion in the wild (Figure 4). Of course, compared to the captive study, the wild one relied on a reduced dataset. However, it was sufficient to verify that yawn contagion can play a role not only at the most basic levels (i.e., OMU) but also at the upper layer of the gelada multi-level social structure (i.e., teams, bands). In wild geladas, yawn contagion was present at significant rates between individuals belonging to different OMUs, with adult males responding more to others’ yawns. The authors suggest that, within the OMU, yawn contagion might foster synchronization between groupmates sharing strong bonds (i.e., grooming), as suggested by the captive approach [90]. At an upper level, beyond the “OMU boundaries”, yawn contagion may help promote the daily activity coordination of different OMUs, as suggested by the wild approach [91]. Yawn contagion is one of the most iconic examples of a behavior that was first detected and documented in captivity and then ‘validated’ in the wild. Without previous captive studies, the wild research on yawn contagion may not have been possible or to ever have been conceived.

An example of generation and validation going in both directions comes from gelada vocal communication. Geladas have long been noted for their complex vocalizations, with some of the early observations coming from captive geladas [92,93]. Working with wild geladas, Gustison et al. [94] showed that gelada vocal complexity (in terms of repertoire size and propensity to combine calls into diverse sequences) was dimorphic and more pronounced in male geladas. Further exploration and experimentation suggested that the function of this vocal complexity was to facilitate bonds between leader males and the multiple females in their unit [95]. Playback experiments even showed that females were more likely to stay in the vicinity of calls from a strange male if those calls were complex and varied. Therefore, the complex calls seem to function to attract females to males. However, it is possible that rather than being inherently attractive, complex sequences were merely less threatening, perhaps because they tend to be produced in relaxed settings. Therefore, to really understand how vocal complexity relates to female preference and sexual selection, we would want to do more controlled choice tests which are not possible with wild animals. Performing these kinds of follow up cognitive validations with captive geladas would make for a more complete study.

## 6. Conclusions

The complementary strengths and weaknesses of research in captive and wild settings makes a combined approach particularly powerful. In the most straightforward application of the complementary approach, one setting can provide what is missing in the other. Wild studies can provide ecological validity that is missing in captivity. Captive studies can provide an observational and experimental control that is lacking in the wild. However, the complementary approach extends beyond this simple filling of gaps in at least three ways. First, captive and wild studies also favor research at different scales that can be synthesized to give a fuller picture of the behavior. This is particularly useful when studying socio-cognitive questions that are themselves directly tied to the scale of social interactions. Secondly, variation in behavior across captive and wild settings is itself a potentially powerful means to explore behavioral flexibility and latent behavioral tendencies. Third, the different settings provide ways of validating and further exploring things that are noticed in the other setting. Observations in the wild might suggest that a particular phenomenon is happening and then captive studies can be used to more robustly probe the behavior. Conversely, observations in captivity might suggest a potential function for a behavior that can be validated in the wild. While we were able to bring together our captive and wild research on geladas to demonstrate these ideas, we would have been better able to do so if we had considered this comparative perspective from the beginning. Therefore, we hope that this manuscript encourages other researchers to think about how to unite captive and wild studies in a more designed and efficient way.

## Data Availability

Not applicable.
